# Soft Tissue Sarcomas: A 16-Year Experience of a Tertiary Referral Hospital in North Jordan

**DOI:** 10.3390/medicina58020198

**Published:** 2022-01-27

**Authors:** Mohammed S. Alorjani, Ismail I. Matalka, Mahmoud A. Alfaqih, Rami A. Jahmani, Belal S. Alsinglawi, Faisal M. Nimri, Mohammad I. Matalka, Samir S. Amr

**Affiliations:** 1 Department of Pathology and Microbiology, Faculty of Medicine, Jordan University of Science and Technology, Irbid 22110, Jordan; imatalka@just.edu.jo; 2 Department of Physiology and Biochemistry, Faculty of Medicine, Jordan University of Science and Technology, Irbid 22110, Jordan; maalfaqih@just.edu.jo; 3 Department of Special Surgery Orthopedic Division, Faculty of Medicine, Jordan University of Science and Technology, Irbid 22110, Jordan; rajahmani@just.edu.jo; 4 School of Computer, Data and Mathematical Sciences, Western Sydney University, Rydalmere, NSW 2116, Australia; b.alsinglawi@westernsydney.edu.au; 5 Internal Medicine Department, Henry Ford Hospital, Detroit, MI 48202, USA; faisalmnimri@gmail.com; 6 King Abdullah University Hospital, Irbid 22110, Jordan; matalka97@gmail.com; 7 Department of Pathology and Laboratory Medicine, Istishari Hospital, Amman 11184, Jordan; samir.amr48@gmail.com

**Keywords:** soft tissue, sarcoma, epidemiology, Jordan

## Abstract

*Background and Objectives*: Sarcomas are rare malignant tumors of mesenchymal origin. Their low prevalence and histological heterogeneity make their diagnosis a challenging task. To the best of our knowledge, the epidemiology of soft tissue sarcomas (STSs) was not well studied in Jordan. This study thus aimed to determine STS epidemiologic trends at King Abdullah University Hospital (KAUH); a tertiary hospital that provides cancer healthcare for 70% of the population in Irbid Governorate, North Jordan. The findings of this study will provide a good reference point of the burden of STSs in Jordan and the Middle East region. *Materials and Methods*: All cases with confirmed STS diagnoses who attended KAUH from January 2003 until December 2018 were included in the initial analysis. Bone sarcomas, gastrointestinal stromal tumors and uterine sarcomas were not included in the study. Information collected from the pathology reports and electronic medical records was used to determine STS prevalence, incidence rate, age and gender distributions, histological types and anatomic location. Cases were reviewed by three pathologists with interest in soft tissue tumors. The findings were compared with literature. *Results*: In total, 157 STS cases were reported (1.9% of cancers diagnosed at KAUH during the 16-year study period). Crude annual incidence rate (IR) per 100,000 person-years ranged from 0.48 in 2015 to 1.83 in 2011 (average = 1.04). Age-standardized IR (ASR)_(World_ _WHO_ _2000–2025)_ was 1.37. Male:female ratio was 1.3:1. Median age was 39 years. Age ranged from <1 year to 90 years. Overall STS rates increased with age. The most common histological types were liposarcoma (19%), rhabdomyosarcoma (17%) and leiomyosarcoma (10%). The most common anatomic location was the extremity (40.1%), followed by the trunk (14.7%), then head and neck (10.8%). *Conclusion*: STSs are rare in North Jordan. A slight increase in their incidence was identified during the study period similar to global trends. The collection of relevant data on established risk factors along with a broader scale evaluation of the epidemiology of STS in the Middle East region is recommended to better evaluate disease burden and trends.

## 1. Introduction

Sarcomas are rare, but serious, malignant tumors of mesenchymal tissue origin. According to recent estimates, sarcomas account for less than 1% of all adult solid malignant tumors. Prevalence of sarcomas is much higher in children where it is estimated that they account for more than 20% of solid cancers [1]. Sarcomas constitute a heterogeneous group of malignancies rather than a single type [2]. According to most recent classifications, there are three main classes of sarcomas corresponding to different clinicopathologic varieties: bone sarcomas, visceral sarcomas that develop in a specific organ (the most typical is gastrointestinal stromal tumors (GIST)), and soft tissue sarcomas (STSs) arising in connective, subcutaneous and other soft tissues, including fat, muscle, nerves, fibrous tissues and blood vessels.

Nearly 70 histological types of sarcomas were recognized by the World Health Organization (WHO), out of which over 50 types belong to STSs [3]. The vast majority of diagnosed sarcomas are STSs, while bone sarcomas make up only about 10% of sarcomas [4]. Numerous risk factors are linked to the development of sarcomas, including age, exposure to radiation, previous malignancy and the genetic background [5]. However, the exact etiology of the majority of sarcomas remains undetermined [6].

The low prevalence rates of sarcomas, their histological heterogeneity and the requirement for proper correlation between clinical–radiological features and pathological findings make their diagnosis a challenging task [7]. In view of the above difficulties, a consensus diagnosis is often difficult to reach [8]. Estimates of disease prevalence in Europe have shown that most frequently observed sarcomas are leiomyosarcoma (19%), liposarcoma (16%) followed by sarcoma not otherwise specified (NOS, 14%) [9]. Prevalence rates are different in the USA and the most common STSs in the USA adult population are undifferentiated pleomorphic sarcomas followed by liposarcomas, and then leiomyosarcoma [8].

Soft tissue sarcomas may occur at any anatomic location. Nevertheless, C47-C49 topography codes (peripheral nerves, autonomic nervous system, retroperitoneum, peritoneum, connective, subcutaneous and other soft tissues) represent the leading location [10], based on the International Classification of Diseases for Oncology ICD-O-3 [11]. About 50% occur in extremities followed in descending order of frequency by the abdominal cavity/retroperitoneum, trunk/thoracic area and head and neck [12]. Certain types of STS have a predilection to occur more frequently in certain parts of the body. For example, liposarcomas and undifferentiated pleomorphic sarcoma are most commonly observed in the lower extremities [8].

Clinical data, biopsy, radiological imaging using CT and/or MRI are all required to reach a definitive diagnosis of STS and to assign a correct grade and stage of the tumor [13]. The staging of STSs, in comparison to cancers in other organs, relies largely on grade [14]. The most widely used STS grading system is the French Federation of Cancer Centers Sarcoma Group (FNCLCC) system [15]. It is divided to three grades and relies on mitotic activity, necrosis, and differentiation. The FNCLCC system is adopted by the WHO and the 8th edition of the American Joint Committee on Cancer *(AJCC) Cancer Staging Manual* because pathology experts found it easy to use and highly reliable in prediction of prognosis [3,16,17].

A recent report was published on primary benign and malignant bone tumors in North Jordan. It found that Ewing sarcoma/Primitive neuroectodermal tumor (PNET), osteosarcoma and chondrosarcoma were the predominant bone sarcoma types [18]. To the best of our knowledge, however, the epidemiology of STSs was not well studied in Jordan and probably not investigated in detail in the Middle East region. This study thus aimed to determine the epidemiologic trends of STSs diagnosed at King Abdullah University Hospital (KAUH). KAUH is a tertiary referral hospital located in Irbid Governorate in North Jordan and provides cancer healthcare for 70% of the Governorate population which accounts for nearly 20% of Jordan’s population [19]. The findings of this study are expected to provide a good reference point of the burden of STSs in Jordan and the Middle East region.

## 2. Materials and Methods

This is a retrospective observational study conducted at KAUH. Histologically proven STS patients who attended and were diagnosed at KAUH during the period from January 2003 to December 2018 were included in the initial analysis. Bone sarcomas, GISTs and female uterine sarcomas were not included in the study. Patients included in the study presented to different clinical departments at KAUH with different complaints, and were assessed by routine blood tests, chest X-ray, ultrasound scan of abdomen, CT scans, MRI scans and surgical material for histological diagnosis. In their pathology reports, method of histological diagnosis (needle core, incisional or excisional biopsy), histological type of STS and grade based on the FNCLCC grading system were described. The pathology reports also included findings of relevant ancillary studies, i.e., immunohistochemical (IHC) marker panels and fluorescence in situ hybridization (FISH) gene rearrangement studies confirming diagnosis/type. Patient pathology reports and electronic medical files were accessed, relevant data were collected, sorted and analyzed to determine the prevalence, incidence rate, age distribution, gender distribution, histological types and anatomic location of STSs. KAUH Cancer Registry was accessed to determine the total number of cancers of different types and origins diagnosed at the hospital during the study period. Ambiguous sarcoma diagnoses and those not following the updated WHO classification scheme were reviewed by three expert pathologists with interests in soft tissue tumors. The population of Irbid Governorate and its gender and age distributions in 2018 and the preceding years of the study period were estimated based on data from the Jordan Department of Statistics [19]. Statistical analysis using the T-Test was performed using SPSS 25 statistical software. The study findings were compared with literature.

## 3. Results

The population served by KAUH for cancer services ranged from 635,460 in 2003 to 1,359,750 in 2018 (average was 944,956). Male percentage based on census data was 51.2% for each of the first 12 years and 51.7% for each of the last 4 years of the study period [19].

**Figure 1:**
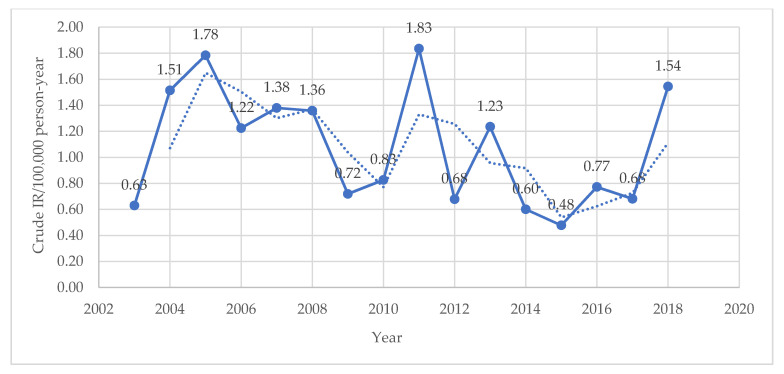
Trend of soft tissue sarcoma crude incidence rate over the period 2003–2018.

In total, 157 cases of soft tissue sarcomas were reported out of 8222 cancer patients admitted and diagnosed at the hospital, accounting for 1.9% of cancers diagnosed at the hospital during the stated period of 16 years (2003–2018). The crude annual incidence rate (IR) per 100,000 person-years ranged from 0.48 in 2015 to 1.83 in 2011 with an average crude IR of 1.04 (95% CI: 0.89–1.21) (Figure 1). The crude IR for males was 1.15, while that for females was 0.92. The age-standardized IR (ASR) for different standard populations were calculated per 100,000 person-years as follow: ASR_(World WHO 2000–2025)_ = 1.37, ASR_(World Segi 1960)_ = 1.30, ASR_(European 2013)_ = 1.93 and ASR_(USA 2000)_ = 1.59. We calculated the ASR_(World Segi 1960)_ in males and females separately and they were 1.43 and 1.18, respectively.

**Figure 2:**
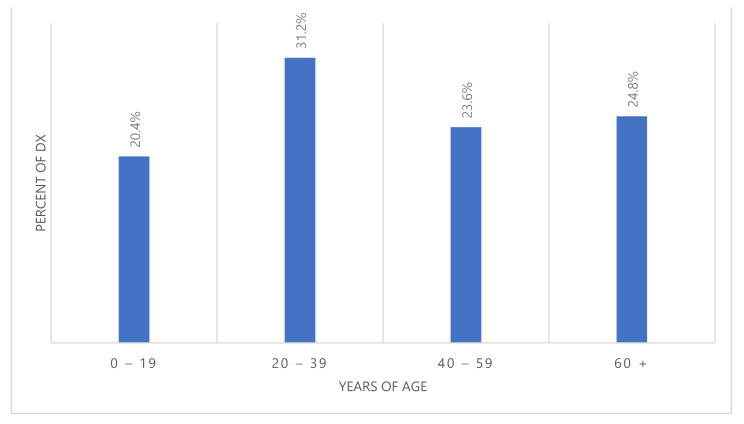
Distribution of ages at diagnosis, 2003–2018.

**Figure 3:**
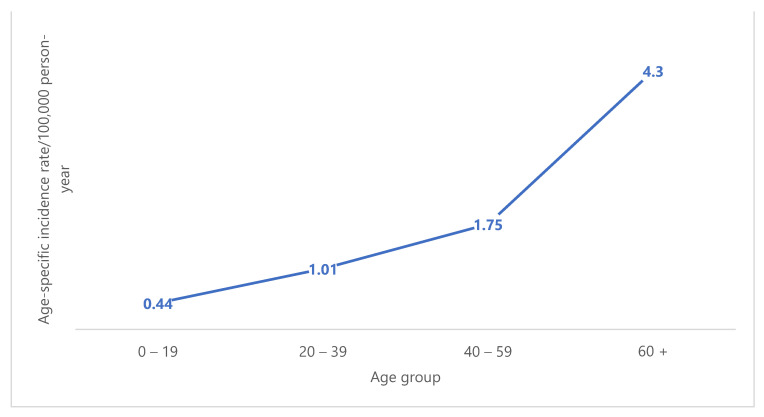
Trend of soft tissue sarcoma incidence rate with age. Age specific incidence rates increased substantially with increasing age.

There was a slight male predominance with 89 males and 68 females (56.7% and 43.3%, respectively) showing a male:female ratio of 1.3:1. The median age was 39 years and age ranged from less than a year to 90 years. Age distribution of cases was as follows: 32 patients (20.4%) were children and adolescents (age group: 0–19), 49 patients (31.2%) were young adults (age group: 20–39), 37 patients (23.6%) were middle-aged adults (age group: 40–59) and the remainder (39 patients; 24.8.%) were older adults aged 60 and over (Figure 2). Overall STS rates increased substantially with increasing age. For both sexes combined, the age-specific IR per 100,000 person-years ranged from 0.44 for individuals aged 0–19 years to 4.3 for those aged 60 years or older (Figure 3).

Overall, 20 histological types of soft tissue sarcoma were reported during the study period. The method of first histological diagnosis was surgical material for all patients (35 by needle biopsy, 44 by incisional biopsy and 78 by excisional biopsy). Diagnoses and histological type determination were aided by IHC marker panels. Ewing sarcoma/PNET and synovial sarcoma diagnoses were confirmed by relevant gene rearrangement FISH tests in the majority of cases performed at other medical centers because this testing modality was not available at our hospital during the study period. The four most common types were liposarcoma (*n* = 30, 19%), rhabdomyosarcoma (*n* = 27, 17%), leiomyosarcoma (*n* = 16, 10%) and malignant peripheral nerve sheath tumor (MPNST) (*n* = 14, 9%). Following in frequency were dermatofibrosarcoma protuberans (DFSP), Ewing sarcoma/PNET and synovial sarcoma which occurred with equal frequencies (*n* = 11, 7% for each). Kaposi sarcoma was next in order (*n* = 6), followed by myxofibrosarcoma and undifferentiated pleomorphic sarcoma (*n* = 5 for each), 4 cases for each of angiosarcoma and low grade fibromyxoid sarcoma, 3 cases for each of clear cell sarcoma and undifferentiated spindle cell sarcoma, 2 cases of epithelioid sarcoma and finally 1 case for each of the following types: alveolar soft part sarcoma, extraskeletal myxoid chondrosarcoma, extraskeletal osteosarcoma and infantile fibrosarcoma. IRs for the four most common histological types of STS are summarized in Table 1.

The most common histological types of STS in males were rhabdomyosarcomas (*n* = 13, 61.9%), Ewing sarcoma/PNET (*n* = 4, 19%), MPNST and leiomyosarcoma (*n* = 1, 4.8% for each) in children and adolescents. The 20–39 year age group showed a high frequency of synovial sarcoma (*n* = 6, 23.1%), liposarcoma (*n* = 5, 19.2%) and DFSP (*n* = 4, 15.4%). The 40–59 year age group showed a high incidence of MPNST (*n* = 5, 26.3%), liposarcoma (*n* = 4, 21%) and leiomyosarcoma (*n* = 3, 15.8%). The older age group (60+ years) showed a high frequency of liposarcoma (*n* = 6. 26%) and leiomyosarcoma (*n* = 2, 8.7%). In females, the most common types were rhabdomyosarcomas (*n* = 7, 63.6%), followed by Ewing sarcoma/PNET, leiomyosarcoma and DFSP with equal frequencies (*n* = 1, 9.1% for each) in children. The 20–39 year age group showed a high frequency of liposarcoma (*n* = 5, 21.8%), MPNST (*n* = 4, 17.4%), followed by Ewing sarcoma/PNET and synovial sarcoma (*n* = 3, 13% for each). The 40–59 year age group showed a high frequency of liposarcoma (*n* = 6, 33.3%), followed by leiomyosarcoma and DFSP (*n* = 2, 11.1% for each). The older age group (60+ years) showed a higher leiomyosarcoma frequency (*n* = 6, 37.5%), followed by liposarcoma (*n* = 4, 25%; Table 2).

**Figure 4:**
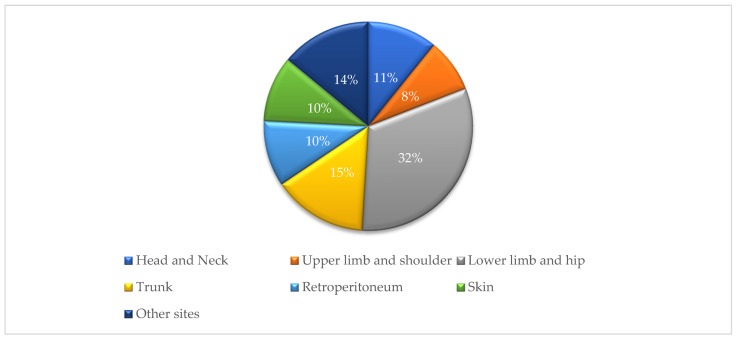
Distribution of soft tissue sarcoma by body site.

The leading topographic locations were connective, subcutaneous and other soft tissues (ICD-O-3 topography code C49); encompassing 91 cases (58%) with additional 30 cases in peripheral nerves, autonomic nervous system, retroperitoneum and peritoneum (ICD-O-3 topography codes C47 and C48), accounting for a total of 121 cases (77%) located in topography codes C47-C49 (Table 3). The most common anatomic location of STS was the extremity, accounting for 40.1% of cases (*n* = 63). In more detail, the lower limb and hip location was predominant (*n* = 50), followed by the trunk (including thorax, abdomen and pelvis; *n* = 23), head and neck (*n* = 17), retroperitoneum and skin which had equal frequencies (*n* = 16 for each), then the upper limb and shoulder location (*n* = 13). The remaining cases were distributed in other sites and organs, including mediastinum and lung (*n* = 8), digestive organs (*n* = 6), urinary bladder and urethra (*n* = 4), along with a single case reported in each of the following organs: breast, testis, adrenal gland and spleen (Figure 4).

Upon analysis of the most frequent STS histological type according to the site of occurrence, liposarcoma was the most common type in the retroperitoneum (75%) and the lower limb and hip (22%) sites; rhabdomyosarcoma was the most frequent type in the trunk (30%) and head and neck (53%) sites; and synovial sarcoma was the most common in the upper limb and shoulder site (38%).

## 4. Discussion

The literature on soft tissue sarcomas in Jordan is relatively scant, with a few series on certain tumors, including rhabdomyosarcoma [20] and synovial sarcoma [21], as well as sporadic case reports on clear cell sarcoma of tendon sheath and aponeurosis [22,23], synovial sarcoma [24,25], liposarcoma [26] and malignant peripheral nerve sheath tumors [27]. The current study is the first in Jordan delineating the spectrum of these uncommon tumor entities, with statistical calculation and estimation of their frequency.

We estimated an average ASR_(World WHO 2000–2025)_ of STS per 100,000 person-years in Irbid, North Jordan, as 1.37. This value lies just below the world values’ range estimated in some older reports, which is from 1.4/100,000 to 5.0/100,000 [6,10,28,29,30,31,32,33]. Small number of STS, along with the use of different criteria and classification schemes of STS in different countries and in various points of time may explain why there are differences in STS IRs among different countries.

In China, a recent study reported ASR_(World Segi 1960)_ of 1.85/100,000 for STSs not including GISTs [34] which is higher than our ASR_(World Segi 1960)_ of 1.3/100,000. In Italy, STS ASR_(World WHO 2000–2025)_ of 1.7/100,000 and ASR_(European 2013)_ of 2.5/100,000 were recently reported [6], which are similar to estimates for Europe as a whole where ASR_(European 2013)_ was 2.3/100,000 [35]. Our rate is below these values (1.37 and 1.93 per 100,000 person-years for ASR_(World WHO 2000–2025)_ and ASR_(European 2013)_, respectively). In the USA, the rate is even much higher, reported by Toro et al. in 2006 to be 5.0/100,000 and more recently to be 3.4/100,000 [32,36]. High STS IRs were also reported in the Netherlands (4.7/100,000) in the period from 1950 to 1988 [37]. These high STS IRs in the USA and the Netherlands were among the highest world rates and may be due to the high frequency of Kaposi sarcoma in addition to the role of applying wider criteria for defining STS in these countries. In Pakistan, a population-based study conducted in Karachi South region reported ASR_(World Segi 1960)_ of 3.3/100,000 males and 2.1/100,000 females over a three-year period (1995–1997); which reflected a high IR and made Karachi a high-risk region for STS with rates close to those reported in the USA and Europe, and higher than those of the rest of the South Asian population [38]. They are also higher than our ASR_(World Segi 1960)_ of 1.43/100,000 males and 1.18/100,000 females.

In the Netherlands, an increasing number of STS new patients was observed over the years [37]. A similar phenomenon was observed in the USA [32]. In Serbia, a study reporting STS incidence in Vojvodina region showed an average ASR_(World WHO 2000–2025)_ of 1.9/100,000 during the observed period (1985–2009) with an increasing tendency over the years with the lowest rate observed in 1990 (1.42/100,000) and the highest in 1997 (2.47/100,000), and an average annual percent change (APC) being 0.77% [10]. In Austria, STS incidence rates are higher than our rates and are equal to 2.4/100,000; similar to those reported in Europe as a whole [33]; however, during the observed period (1984–2004), no change was identified in the number of new STS patients. Similar to the Netherlands’ and Serbia’s findings, our study showed an increase in IR over the years during the observed study period (2003–2018) where the crude IR per 100,000 person-years for both sexes combined ranged from 0.63 in 2003 to 1.54 in 2018. However, this increase was not steady because we observed oscillations in rates and the year 2011 reported the highest rate (1.83/100,000) with the lowest rate observed in 2015 (0.48/100,000).

The finding that nearly every second STS patient in the current study is younger than 40 years may be partly explained by the high frequency of rhabdomyosarcoma in this study of almost 17% ranking second, which is mainly a malignancy of childhood and adolescence [33]. This value is higher than in Serbia where 20% of patients were younger than 40 [10]. Age-specific IRs depicted a substantial increase in STS rates with advancing age in this study, and a similar trend was noted in some literature reports. For example, a rapid increasing trend was reported in Europe until ages 40–50 years with little variation thereafter [39] and in the USA, an increase in annual IR with age was reported, rising from 0.9/100,000 in children younger than 10 years of age to 18.2/100,000 in adults over 70 years old [40]. Similar to the current study, STS is slightly more common in males than in females in the USA [32] with a male:female ratio slightly over 1, but the opposite trend was reported in Serbia and Austria where male:female ratio was ≤0.8 [10,33].

Upon analysis of histological types, we found that 19% of STSs were liposarcoma, followed by rhabdomyosarcoma (17%), leiomyosarcoma (10%), MPNST (9%) and 7% for each of synovial sarcoma, Ewing sarcoma/PNET and DFSP. Significant differences in frequency of histological types were noted among studies from different countries. However, the current study showed that the most common sarcomas were somehow similar to those reported in a good number of reports in the recent literature with some variation in order of frequency, probably related to difference in age group structure of the population. In Japan [41], for example, liposarcoma was the most frequent STS similar to the current study, and accounted for 32.5% of cases; however, the order of frequency of different liposarcoma subtypes was different (well-differentiated, followed by myxoid-round cell, then dedifferentiated subtype in the Japanese study versus myxoid/round cell, followed by dedifferentiated, then well-differentiated subtype in the current study). The second in order in Japan was undifferentiated pleomorphic sarcoma (19.5%), followed by leiomyosarcoma (6.5%), myxofibrosarcoma (5.9%), synovial sarcoma (5.8%) and MPNST (4.5%). Rhabdomyosarcoma, which was the second in frequency in our study (17%), ranked seventh accounting for only 2.5% of STSs in Japan, and Ewing sarcoma/PNET was even much less frequent (1.9%). Such significantly lower proportions of rhabdomyosarcoma and Ewing sarcoma/PNET in Japan compared to the current study are probably related to these being mainly malignancies of childhood and adolescence [33] and that the Japanese population is a super-aging society [42], compared to Jordan’s population. Although life expectancy in Jordan is gradually increasing and it ranged during the study period from 72.256 in 2003 to 74.405 in 2018 with a 0.18% annual increase in the last few years [43], Jordan population statistics data showed that in 2015, for example, only 3.7% of the population were at 65 years or older and 34.3% younger than 15 years [19]. Two other studies, one from Europe covering three European regions [39] and the second from Southern India [12] showed, similar to our findings, that liposarcoma was the most common STS (26.2%, 17.6% in the two studies, respectively); however, second rank was for leiomyosarcoma in both studies (16.1% and 15.7%, respectively). DFSP ranked third in the European study (10.1%) compared to the current study (fifth order at 7%) which is in the range of DFSP frequency (6.2–9.5%) in many world reports [28,32,37]. Ewing sarcoma/PNET came third in order (13.7%) in the Southern India report in comparison with the current study where it shared fifth rank with DFSP and synovial sarcoma. In the Southern India report, rhabdomyosarcoma ranked fourth in frequency (11.8%), compared to our study in which it ranked second at 17%.

On the other hand, many other studies from different parts of the world showed significantly different frequencies of the common STSs in comparison with the current study. For instance, undifferentiated sarcoma NOS was the most frequent STS in recent studies from Austria, Serbia, China and Italy [6,10,33,34] ranging from nearly a quarter to 36% of STSs across these studies. In some other reports, leiomyosarcoma ranked first in frequency [9,31,32,37] ranging from 18% to one every fourth patient. The Swedish study by Gustafson was an exception to these observations where malignant fibrous histiocytoma (MFH) was diagnosed with the greatest frequency at 41% [29], but this is probably because the study was conducted in 1994 covering a period ending in 1989 when MFH was still adopted in STS classification before the 2002 WHO classification. Thereafter, MFH was considered an obsolete term and was abandoned. In the current study, expert sarcoma pathologists reviewed the cases, especially those with ambiguous or obsolete diagnoses (*n* = 19). The diagnoses were amended in three cases because the obsolete class of MFH was used in the original reports; two of which were reclassified as dedifferentiated liposarcoma and one was renamed undifferentiated pleomorphic sarcoma. In nine cases, the final diagnoses were not genuinely changed but were reformulated to accurately follow the WHO classification scheme. In the remaining seven reviewed cases, the diagnoses remained as they were in the original reports.

Kaposi sarcoma accounted for nearly 4% of STSs in the current study, a figure which was in the lower end of literature values and close to the 1% of Austria [33] and 2% of Serbia [10] in comparison with 15% in the Netherlands, USA and Switzerland [28,31,37]. GISTs and female uterine sarcomas were not included in this study.

In the current study, the leading topographic locations were connective, subcutaneous and other soft tissues (ICD-O-3 topography code C49); encompassing 91 cases (58%) with an additional 30 cases in peripheral nerves, autonomic nervous system, retroperitoneum and peritoneum (ICD-O-3 topography codes C47 and C48), producing a total of 121 cases (77%) located in topography codes C47-C49 similar to most other studies [10,32,33]. Yang et al. reported in China, however, the C49 topography code in only 22.6% of cases [34], but this is probably because of the inclusion of GIST in their study leading to a higher contribution (20.6%) of digestive tract (ICD-O-3 topography codes C15-C26) location compared to those studies.

The most common anatomic location of STSs in this study was the extremity (40.1%), with the lower extremity and hip location representing a predominant site (31.8%) similar to many published reports from different world regions. For example, reports from Italy, Karachi South—Pakistan, Osaka—Japan, North Netherlands and USA showed extremity involvement in 55%, 49%, 43%, 45% and 40% of cases, respectively, with all these studies reporting higher incidence in the lower limb than in the upper limb [6,38,44,45,46].

Similar to our findings, liposarcoma was the most frequent STS type in the lower extremity in North Italy [6]. However, it was also the most common type in the trunk, contrary to the results of the current study in which rhabdomyosarcoma was dominant at this site. Moreover, in North Italy, leiomyosarcoma and fibrosarcoma were the most frequent types in the head and neck and upper extremity sites, respectively, in comparison with the current study in which rhabdomyosarcoma and synovial sarcoma dominated at these two sites, respectively.

The main strength of this study is that it is, to the best of four knowledge, the first to investigate the epidemiology of these rare cancers, STSs of different types, in Jordan and is probably the first to investigate such detailed epidemiological parameters of STSs in the Middle East region. A second strength is that the histopathological diagnoses offered over the study period were thoroughly reviewed by expert sarcoma pathologists to correct any erroneous diagnosis and to follow the current WHO classification scheme in the study. In terms of limitation, our study is based on the practice in a medical center (KAUH) and is not population-based covering only a proportion of the population who are provided with health care in this center. This precluded our ability to accumulate a sufficient number of cases of STS at a single medical center for statistical analysis of this rare cancer. However, KAUH is a big tertiary referral center and the main cancer care center in North Jordan, providing care for a good proportion of the population of Jordan; therefore, we believe that the study reasonably reflects STS data of Jordan and will act as an important core step for future work on this rare but serious cancer type in collaboration with other cancer centers in our country and the region.

## 5. Conclusions

STSs are rare in North Jordan with incidence rates below the world’s values’ range. A slight increase in the incidence of STS was identified during the study period similar to global trends. The collection of relevant data on established risk factors along with a broader scale evaluation of the epidemiology of STS through collaboration with other cancer centers in Jordan and the Middle East region is recommended to better evaluate disease burden and trends.

## Figures and Tables

**Table 1:** Incidence rates (IR) for the four most common histological types of soft tissue sarcoma.

Histological Type of Sarcoma	*n*	IR	95% CI
Liposarcoma	30	0.1984	0.14–0.28
Rhabdomyosarcoma	27	0.1786	0.12–0.26
Leiomyosarcoma	16	0.1058	0.06–0.17
MPNST	14	0.0926	0.05–0.16

MPNST: malignant peripheral nerve sheath tumor.

**Table 2:** Histological type distribution of soft tissue sarcoma by gender and age group (% data). DFSP: dermatofibrosarcoma protuberans. LMS: leiomyosarcoma. LPS: liposarcoma. MPNST: malignant peripheral nerve sheath tumor. RMS: rhabdomyosarcoma. PNET: primitive neuroectodermal tumor. SS: synovial sarcoma.

Gender		Relative Frequency	LPS	RMS	LMS	MPNST	DFSP	Ewing Sarcoma/PNET	SS	Others
Male	0–19 y	23.6		61.9	4.8	4.8		19		9.5
	20–39 y	29.2	19.2	7.7	7.7	7.7	15.4	3.8	23.1	15.4
	40–59 y	21.4	21		15.8	26.3	5.3	5.3	5.3	21
	60+ y	25.8	26	8.7	4.4	4.4	4.4			52.2
Female	0–19 y	16.2		63.6	9.1		9.1	9.1		9.1
	20–39 y	33.8	21.8	8.7		17.4	8.7	13	13	17.4
	40–59 y	26.5	33.3		11.1	5.6	11.1	5.6	5.6	27.8
	60+ y	23.5	25	6.25	37.5					31.25

**Table 3:** Topography distribution of soft tissue sarcoma.

ICD-O-3 Topography Code	Location	*n* (%)
C15-C26	Digestive organs	5 (3.18)
C30-C39	Respiratory system and intrathoracic organs	7 (4.46)
C42	Hematopoietic and reticuloendothelial system	1 (0.64)
C44	Skin	16 (10.2)
C47	Peripheral nerves and autonomic nervous system	14 (8.9)
C48	retroperitoneum and peritoneum	16 (10.2)
C49	Connective, subcutaneous and other soft tissues	91 (58)
C50	Breast	1 (0.64)
C60-C63	Male genital organs	1 (0.64)
C64-C68	Urinary tract	4 (2.5)
C73-C75	Endocrine glands	1 (0.64)

## Data Availability

Not applicable.

## References

[1] SEER*Stat Database: Incidence—SEER 9 Regs Research Data, November 2010 Sub (1973–2008) <Katrina/Rita Population Adjustment>—Linked To County Attributes—Total U.S., 1969–2009 Counties, National Cancer Institute, DCCPS, Surveillance Research Program, Cancer Statistics Branch, released April 2011, based on the November 2010 submission. Surveillance, Epidemiology, and End Results (SEER) Program. http://www.seer.cancer.gov.

[2] Lahat G., Lazar A., Lev D. (2008). Sarcoma epidemiology and etiology: Potential environmental and genetic factors. Surg. Clin. N. Am..

[3] WHO Classification of Tumours Editorial Board (2020). Soft Tissue and Bone Tumors.

[4] Burningham Z., Hashibe M., Spector L., Schiffman J.D. (2012). The epidemiology of sarcoma. Clin. Sarcoma Res..

[5] Penel N., Grosjean J., Robin Y.M., Vanseymortier L., Clisant S., Adenis A. (2008). Frequency of certain established risk factors in soft tissue sarcomas in adults: A prospective descriptive study of 658 cases. Sarcoma.

[6] Fabiano S., Contiero P., Barigelletti G., D’Agostino A., Tittarelli A., Mangone L., Bisceglia I., Bongiorno S., De Lorenzis L.E., Mazzoleni G. (2020). Epidemiology of Soft Tissue Sarcoma and Bone Sarcoma in Italy: Analysis of Data from 15 Population-Based Cancer Registries. Sarcoma.

[7] Ray-Coquard I., Montesco M.C., Coindre J.M., Dei Tos A.P., Lurkin A., Ranchère-Vince D., Vecchiato A., Decouvelaere A.V., Mathoulin-Pélissier S., Albert S. (2012). Sarcoma: Concordance between initial diagnosis and centralized expert review in a population-based study within three European regions. Ann. Oncol..

[8] American Cancer Society (2021). Cancer Facts & Figures 2021.

[9] Saltus C.W., Calingaert B., Candrilli S., Lorenzo M., D’yachkova Y., Otto T., Wagner U., Kaye J.A. (2018). Epidemiology of Adult Soft-Tissue Sarcomas in Germany. Sarcoma.

[10] Dugandzija T., Mikov M.M., Solajic N., Nikolin B., Trifunovic J., Ilic M. (2014). Increasing frequency of soft tissue sarcomas in Vojvodina—comparison with the literature. Asian Pac. J. Cancer Prev..

[11] Fritz A., Percy C., Jack A., Shanmugaratnam K., Sobin L., Parkin D.M., Whelan S. (2013). International Classification of Diseases for Oncology (ICD-O).

[12] Gupta A., Rao H.K., Gupta S. (2009). The incidence of soft tissue sarcoma in Dakshina Kannada: Study in a District Government Hospital. Indian J. Surg..

[13] Gaynor J.J., Tan C.C., Casper E.S., Collin C.F., Friedrich C., Shiu M., Hajdu S.I., Brennan M.F. (1992). Refinement of clinicopathologic staging for localized soft tissue sarcoma of the extremity: A study of 423 adults. J. Clin. Oncol..

[14] Eilber F.C., Brennan M.F., Eilber F.R., Dry S.M., Singer S., Kattan M.W. (2004). Validation of the postoperative nomogram for 12-year sarcoma-specific mortality. Cancer.

[15] Coindre J.M. (2006). Grading of soft tissue sarcomas: Review and update. Arch. Pathol. Lab. Med..

[16] Amin M.B., Edge S.B., Greene F.L., Byrd D.R., Brookland R.K., Washington M.K., Gershenwald J.E., Compton C.C., Hess K.R., Sullivan D.C. (2017). AJCC Cancer Staging Manual.

[17] Guillou L., Coindre J.M., Bonichon F., Nguyen B.B., Terrier P., Collin F., Vilain M.O., Mandard A.M., Le Doussal V., Leroux A. (1997). Comparative study of the National Cancer Institute and French Federation of Cancer Centers Sarcoma Group grading systems in a population of 410 adult patients with soft tissue sarcoma. J. Clin. Oncol..

[18] Bashaireh K.M., Alorjani M., Jahmani R.A., Al Khateeb A., Nimri F., Al-Ebbini M.A., Ababneh A.R.M. (2021). Primary Bone Tumors in North of Jordan. J. Epidemiol. Glob. Health.

[19] Population—Jordan Department of Statistics. http://dosweb.dos.gov.jo.

[20] Mustafa M., Al-jarrah O., Hamoury M.K., Alhiwat S., Al-Hassanat O. (2016). Pediatric rhabdomyosarcoma: A 7-year experience at King Hussein Medical Center. J. R. Med. Serv..

[21] Yaser S., Salah S., Al-Shatti M., Abu-Sheikha A., Shehadeh A., Sultan I., Salem A., Sughayer M., Al-Loh S., Al-Mousa A. (2014). Prognostic factors that govern localized synovial sarcoma: A single institution retrospective study on 51 patients. Med. Oncol..

[22] Amr S.S., Farah G.R., Muhtaseb H.H., Al-Hajj H.A., Levene A. (1984). Clear cell sarcoma: Report of two cases with ultrastructural observations and review of the literature. Clin. Oncol..

[23] Abou Chaar M.K., Jaber O.I., Asha W., Abdel Al S. (2020). Novel Double Central Ray Amputation of the Third and Fourth Digits: Case Report and Literature Review. Case Rep. Oncol..

[24] Amr S.S., Shihabi N.K., Al Hajj H. (1984). Synovial sarcoma of the esophagus. Am. J. Otolaryngol..

[25] Salah S., Al-Ibraheem A., Daboor A., Al-Hussaini M. (2013). Synovial sarcoma presenting with huge mediastinal mass: A case report and review of literature. BMC Res. Notes.

[26] Muhsen B.A., Ghzawi A., Fares A.S., Al-Hussaini M., Salah S. (2021). Metastatic myxoid liposarcoma of the brain: A case report and review of the literature. Future Sci. OA.

[27] Abdel Al S., Abou Chaar M.K., Asha W., Al-Najjar H., Al-Hussaini M. (2020). Fungating malignant peripheral nerve sheath tumor arising from a slow-growing mass in the forearm: A case report and review of the literature. J. Med. Case Rep..

[28] Ross J.A., Severson R.K., Davis S., Brooks J.J. (1993). Trends in the incidence of soft tissue sarcomas in the United States from 1973 through 1987. Cancer.

[29] Gustafson P. (1994). Soft tissue sarcoma. Epidemiology and prognosis in 508 patients. Acta Orthop. Scand. Suppl..

[30] Storm H.H. (1994). Cancers of the soft tissues. Cancer Surv..

[31] Levi F., La Vecchia C., Randimbison L., Te V.C. (1999). Descriptive epidemiology of soft tissue sarcomas in Vaud, Switzerland. Eur. J. Cancer.

[32] Toro J.R., Travis L.B., Wu H.J., Zhu K., Fletcher C.D., Devesa S.S. (2006). Incidence patterns of soft tissue sarcomas, regardless of primary site, in the surveillance, epidemiology and end results program, 1978–2001: An analysis of 26,758 cases. Int. J. Cancer.

[33] Wibmer C., Leithner A., Zielonke N., Sperl M., Windhager R. (2010). Increasing incidence rates of soft tissue sarcomas? A population-based epidemiologic study and literature review. Ann. Oncol..

[34] Yang Z., Zheng R., Zhang S., Zeng H., Li H., Chen W. (2019). Incidence, distribution of histological subtypes and primary sites of soft tissue sarcoma in China. Cancer Biol. Med..

[35] Rare Cancer Network in Europe. http://www.rarecarenet.eu.

[36] National Cancer Institute, Surveillance Research Program, National Cancer Institute, Bethesda, MA, USA, 2016. https://seer.cancer.gov/statfacts/html/soft.html.

[37] Schuurman B., Meyer S., Cuesta M.A., Nauta J.J. (1992). Stijgende frequentie van weke-delensarcomen in Nederland [Increasing frequency of soft tissue sarcomas in The Netherlands]. Ned. Tijdschr. Geneeskd..

[38] Bhurgri Y., Bhurgri H., Pervez S., Kayani N., Usman A., Bashir I., Bhurgri A., Hasan S.H., Zaidi S.M. (2008). Epidemiology of soft tissue sarcomas in Karachi South, Pakistan (1995–1997). Asian Pac. J. Cancer Prev..

[39] Mastrangelo G., Coindre J.M., Ducimetière F., Dei Tos A.P., Fadda E., Blay J.Y., Buja A., Fedeli U., Cegolon L., Frasson A. (2012). Incidence of soft tissue sarcoma and beyond: A population-based prospective study in 3 European regions. Cancer.

[40] Ferrari A., Sultan I., Huang T.T., Rodriguez-Galindo C., Shehadeh A., Meazza C., Ness K.K., Casanova M., Spunt S.L. (2011). Soft tissue sarcoma across the age spectrum: A population-based study from the Surveillance Epidemiology and End Results database. Pediatr. Blood Cancer.

[41] Ogura K., Higashi T., Kawai A. (2017). Statistics of soft-tissue sarcoma in Japan: Report from the Bone and Soft Tissue Tumor Registry in Japan. J. Orthop. Sci..

[42] (2021). Statistics Bureau of Japan. https://www.stat.go.jp.

[43] The World Bank. https://data.worldbank.org/indicator/SP.DYN.LE00.IN?locations=JO.

[44] Tsujimoto M., Aozasa K., Ueda T., Sakurai M., Ishiguro S., Kurata A., Ono K., Matsumoto K. (1988). Soft tissue sarcomas in Osaka, Japan (1962–1985): Review of 290 cases. Jpn. J. Clin. Oncol..

[45] Nijhuis P.H., Schaapveld M., Otter R., Molenaar W.M., van der Graaf W.T., Hoekstra H.J. (1999). Epidemiological aspects of soft tissue sarcomas (STS)—consequences for the design of clinical STS trials. Eur. J. Cancer.

[46] Hui J.Y. (2016). Epidemiology and Etiology of Sarcomas. Surg. Clin. N. Am..

